# The Effect of UV Treatment on Surface Contact Angle, Fibroblast Cytotoxicity, and Proliferation with Two Types of Zirconia-Based Ceramics

**DOI:** 10.3390/ijerph191711113

**Published:** 2022-09-05

**Authors:** Vygandas Rutkunas, Rokas Borusevicius, Evaldas Balciunas, Urte Jasinskyte, Milda Alksne, Egidijus Simoliunas, Stefan Zlatev, Vasilena Ivanova, Virginija Bukelskiene, Eitan Mijiritsky

**Affiliations:** 1 Institute of Odontology, Faculty of Medicine, Vilnius University, 03101 Vilnius, Lithuania; 2 Institute of Biochemistry, Life Sciences Center, Vilnius University, 03101 Vilnius, Lithuania; 3 CAD/CAM Center of Dental Medicine at the Research Institute, Medical University of Plovdiv, 4000 Plovdiv, Bulgaria; 4 Oral Surgery Department, Faculty of Dental Medicine, Medical University of Plovdiv, 4000 Plovdiv, Bulgaria; 5 Head and Neck Maxillofacial Surgery, Tel-Aviv Sourasky Medical Center, Department of Otolaryngology, Sackler Faculty of Medicine, Tel-Aviv University, Tel-Aviv 699350, Israel

**Keywords:** surface roughness, water contact angle, fibroblast proliferation, zirconia, UV photofunctionalization

## Abstract

UV photofunctionalization of Zirconia-based materials for abutment fabrication is a promising approach that might influence the formation of a sound peri-implant seal, thus promoting long-term soft and hard tissue implant integration. This study aimed to evaluate the effect of UV treatment of test specimens made by two different ZnO_2_-based ceramic materials on the hydrophilicity, cell cytotoxicity, and proliferation of human gingival fibroblasts (HGFs). Two Zirconia-based materials, high-translucent and ultra-translucent multi-layered Zirconia (Katana, Kuraray Noritake, Japan), were used to prepare a total of 40 specimens distributed in two equally sized groups based on the material (*n* = 20). The same surface finishing protocol was applied for all specimens, as suggested by the manufacturer. Half the specimens from each group were treated with UV-C light for 48 h. Water contact angle (WCA), fibroblast cytotoxicity, and proliferation were investigated. The WCA values for the high-translucent Zirconia ranged from 69.9° ± 6.4° to 73.7° ± 13.9° for the treated/non-treated specimens and from 79.5° ± 12.8° to 83.4° ± 11.4° for the ultra-translucent multi-layered Zirconia, respectively. However, the difference was insignificant (F(16) = 3.50, *p* = 0.292). No significant difference was observed for the fibroblast cytotoxicity test. The results for proliferation revealed a significant difference, which was material-dependent (F(8) = 9.58, *p* = 0.005). We found that UV surface photofunctionalization of ZrO_2_-based materials alters the human gingival fibroblast cell viability, which might produce favourable results for cell proliferation.

## 1. Introduction

Recently, dental implants have become a widespread treatment option for restoring partially and fully edentulous patients, with an ever-growing body of scientific evidence supporting their numerous benefits compared to a more conventional approach. After overcoming the challenges of osseointegration, most studies currently focus on peri-implant soft tissues, proving that their quantity and quality are essential for bone stability around dental implants [1]. Soft tissue integration in the transmucosal zone of implant abutments is a vital functional and biological parameter for supporting the peri-implant tissues, improving aesthetics, ensuring soft tissue seal against microorganisms, and preserving crestal bone loss, ultimately increasing the longevity of the restoration [2].

Various materials are used for abutment fabrication in contemporary implant dentistry, incorporating all major classes: metals, polymers, and ceramics. All of them have unique advantages and disadvantages [3]. Among the different abutment materials, the ones based on ZrO_2_ offer several distinct benefits. Zirconia has exceptional strength and fracture resistance, which makes it an optimal choice even in biomechanically challenging situations. Furthermore, it is corrosion resistant and has excellent aesthetic properties [4].

The final metal-free implant-supported restoration colour strongly depends on its design and the material used for abutment fabrication [5]. The optical properties of Zirconia are favourable for the restorations’ final colour and positively influence the shade of the peri-implant soft tissues when an appropriate biotype is present [6,7]. Based on colour, Zirconia can be divided into two major classes—white and pre-coloured—also referred to as multi-layered [8]. Furthermore, ZrO_2_-based abutments show better outcomes than other ceramic materials [9].

Another essential advantage of Zirconia-based abutments is their excellent biocompatibility [10]. Studies suggest a similar soft tissue blood flow around Zirconia-based abutments and natural teeth and improved microcirculation compared to other abutment materials, which could be advantageous for the immune response [11,12]. Furthermore, the inflammation infiltrates around ZrO_2_ abutments are lower than around titanium ones, suggesting a better soft tissue seal with decreased bacterial invasion and reduced crestal bone loss during the first year [4,13].

Several factors can influence the soft tissue response to Zirconia-based abutments. Surface microtopography and hydrophilicity play a significant role and have been extensively studied previously [14,15,16,17]. Surface roughness accounts for two important characteristics of human gingival fibroblast (HGF) proliferation and attachment—time and orientation of the fibres to the abutment surface. Results reported by different studies support the thesis that a moderately rough surface, between 1 and 2 micrometres, defined as nanotubes or directional grooves, creates optimal conditions for HGF attachment and soft tissue seal formation [18].

Hydrophilicity, measured as a water contact angle (WCA) function, is considered among the main factors affecting protein absorption and human gingival fibroblasts’ cellular attachment to implant abutments [19,20]. Generally, a lower contact angle promotes fibroblast attachment [20]. ZrO_2_-based ceramics used for abutment fabrication show a defined hydrophobic behaviour, with contact angles sometimes exceeding the hydrophilic threshold of 40° by more than twice [21].

Different surface modifications have been proposed to create more favourable conditions between the implant abutment and its surrounding soft tissues [17]. Among them, applying UV photofunctionalization to Zirconia-based materials shows promising results [22,23]. The method is easy to perform chairside and brings distinct advantages, such as a substantial decrease in surface carbon content and increased wettability, proliferation, and attachment of cell structures [24,25]. However, the literature on hydrophilicity and HGF-related factors of UV-treated Zirconia-based materials used for implant abutment fabrication is inconclusive. Furthermore, only a few commercially available materials have been tested previously.

This study aimed to evaluate the effect of UV treatment of test specimens made of two different ZrO_2_-based ceramic materials on the hydrophilicity, cell cytotoxicity, and proliferation of human gingival fibroblasts (HGFs).

## 2. Materials and Methods

The current study was approved by the ethical committee protocol #158200-16-860-369. Informed consent was obtained from the tissue donor.

Two materials for abutment fabrication were selected for the study (see Table 1).

The sample size was determined by analysing the available literature of relevant articles published on the topic. Twenty test specimens (*n* = 20) were produced from each material. These were further randomly allocated to one of the groups based on surface treatment, with ten per material in each group (*n* = 10).

**Figure 1:**
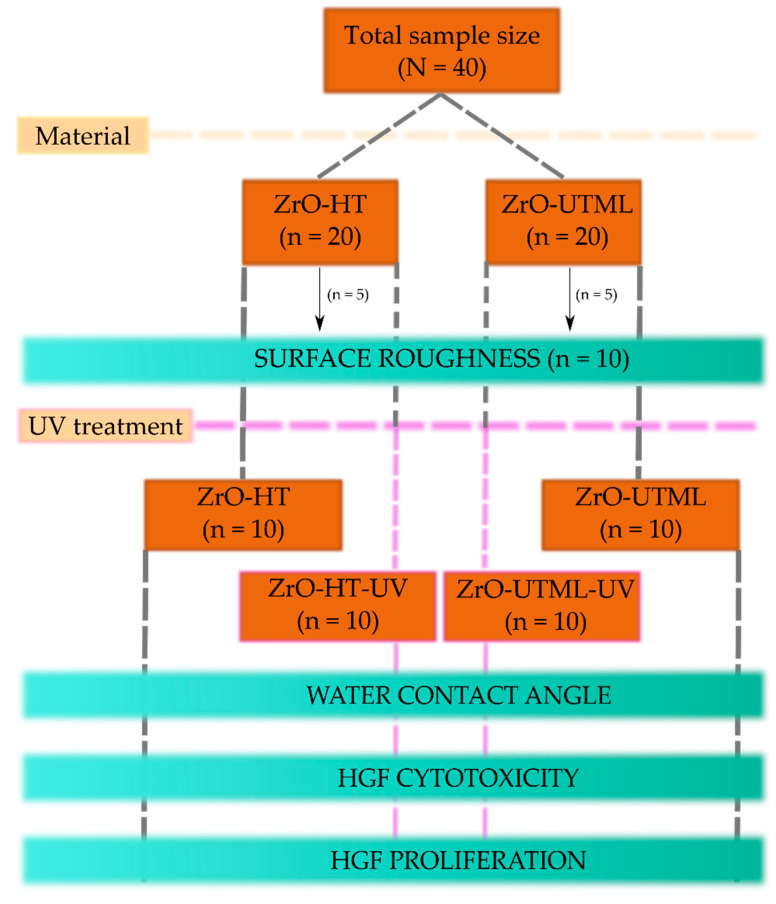
Study design scheme.

The study design is demonstrated in Figure 1 and the following subsections. The different steps are described in the following subsections.

### 2.1. Manufacturing of Specimens and Surface Finishing Protocol

All specimens were milled using a computer numeric controlled machine (Vhf CAM 5-S1 Impression, vhf camfacture AG, Ammerbuch, Germany) into cylinders, which were subsequently cut into tablets. Cutting was performed using a saw microtome (Leica SP 1600, Leica Biosystems Nussloch GmbH, Nußloch, Germany) with a water-cooling agent before sintering.

**Figure 2:**
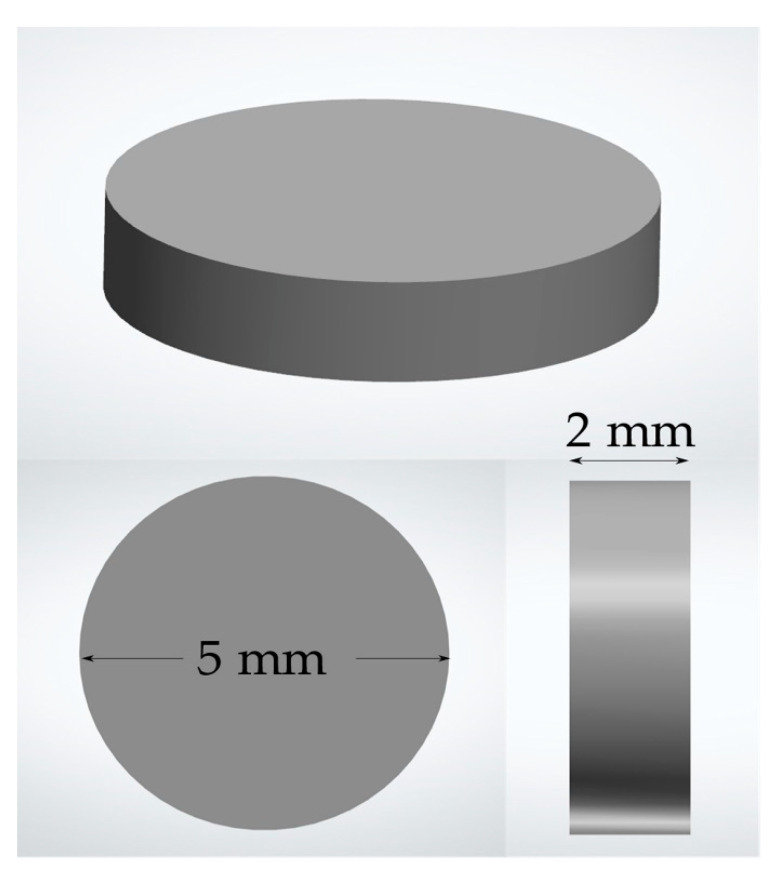
Dimensions of specimens used in the study.

The specimens were cut larger to compensate for the shrinkage coefficient. The exact dimensions of the specimens after sintering were 2 mm in height and 5 mm in diameter (Figure 2). The sintering procedures were performed according to the manufacturer’s instructions. All surfaces were polished according to protocols suggested by the manufacturer (Table 2). Specimens were polished each time following the exact same protocol before every separate experiment in the study.

Specimens were soaked in “Decon” solution (Decon Laboratories™ Decon 90™, Decon Laboratories Limited, Sussex, England, Fisher Scientific, Portsmouth, NH, USA) and put on a laboratory tumbling table (Mini-Tumbling Table WT17, 25 rpm, angle of inclination 5°/10°, Biometra GmbH, Göttingen, Germany) for 24 h. Then, they were rinsed with tap water 20 times and subsequently with distilled water 10 times. Finally, specimens were soaked in 70% ethanol for 24 h. After washing procedures, each side of the specimens was air-dried at room temperature for 24 h. The same protocol was repeated after every specimen polishing session just before the following experiment.

### 2.2. Profilometry

**Figure 3:**
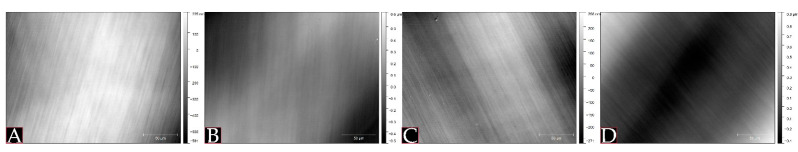
Profilometry images for both materials included in the study based on the location: (**A**) ZrO-HT centre, (**B**) ZrO-HT periphery, (**C**) ZrO-UTML centre, (**D**) ZrO-UTML periphery.

Surface mean roughness (Ra) was measured using an optical profiler topographic analysis (PLμ 2300, Sensofar, Sensofar Group, Barcelona, Spain) with a confocal objective of 50×/0.8 A with FOV 255 × 191 μm (Nikon Lu Plan, Nikon Metrology NV, Leuven, Belgium). Five specimens from each group were randomly selected. Three images of surface areas (two areas were chosen at random on a periphery and one in the centre, Figure 3) on every specimen were taken. The images were processed, and Ra values were computed using Gwyddion Software (Czech Metrology Institute, Jihlava, Czech Republic).

### 2.3. UV Photofunctionalization

Ten specimens per material group were selected randomly and subjected to UV surface photofunctionalization. The specimens were treated with a UV-C light system (Sylvania G15W T8 lamps, Feilo Sylvania Group, Shanghai Feilo Acoustics Co., Budapest, Hungary) with a peak wavelength of 253.7 nm for 48 h at a distance of 12 cm. The procedure resulted in irradiance of 3.49 mW/cm^2^ and was repeated between different tests. Photofunctionalization was repeated before each experiment for UV-treated groups.

### 2.4. Water Contact Angle Measurement

Hydrophilicity was assessed by measuring the WCA in this study. The specimens were set in a Krüss EasyDrop (KRÜSS GmbH, Hamburg, Germany) system. The specimen chamber temperature was kept constant at 21 °C using a LabTech H50-500 water cooler (LabTech Srl, Sorisole BG, Italy). Deionised water droplets (16 Ω, 2 μL) were placed on the specimens. After 10 s, pictures were taken and analysed using Krüss software (KRÜSS GmbH, Hamburg, Germany). The data for each measurement were recorded as the mean value of the measurements obtained from the two sides of each water droplet. Five specimens were measured from each group. The results were presented as mean ± standard deviation.

### 2.5. Cell Culturing

Primary human gingival fibroblast (HGF) culture was used in this study. Fibroblasts were received using the technique described in previous research from a healthy patient during periodontal surgical treatment (approved by the National Bioethics Committee, No 158200-16-860-369) [15]. HGF monolayer was cultivated in an IDMEM (Iscove’s Modified Dulbecco’s Medium; Gibco, Thermo Fisher Scientific, Waltham, MA, USA) with 10% FCS (foetal calf serum; Gibco, Thermo Fisher Scientific, Waltham, MA, USA) and antibiotics (penicillin 100 VV/mL, and streptomycin 100 μg/mL; Gibco, Thermo Fisher Scientific, Waltham, MA, USA) in 50 mL polystyrene flasks (Greiner, Greiner Bio-One GmbH, Frickenhausen, Germany). Fibroblasts from 6 to 12 passages were used. HGF were cultivated in the incubator (HeracellTM 150i, Thermo Fisher Scientific, Waltham, MA, USA) at 37 °C, 5% CO_2_, 95% H_2_O. Passaging was performed 2 times per week.

### 2.6. HGF Cytotoxicity and HGF Proliferation Assessment

This study used 96-well plates (Greiner, Greiner Bio-One GmbH, Frickenhausen, Germany). A suspension of HGF (30 × 10^3^ cells/mL) was prepared and poured into the plate wells with the specimens, 200 μL each, to evaluate the cytotoxicity and proliferation. For the HGF cytotoxicity evaluation, three specimens of every group were used and the number of viable cells on the specimens and the plastic control surface was registered at 12 h. The experiment was repeated three times. For the HGF proliferation assessment, three specimens from each group were used per time point (registered at 24, 48, and 72 h), respectively, and the experiment was repeated three times.

The viable cell amount was evaluated using the MTT (3-(4,5-dimetiltiazol-2-il)-2,5-diphenyltetrazolium bromide) method. At each time point, the cell growth medium was cautiously eliminated from every well, and each was filled with 100 μL of MTT (Merck Chemicals, Merck KGaA, Darmstadt, Germany), 1 mg/mL prepared in phosphate-buffered saline. MTT was removed after one hour of incubation at 37 °C, 5% CO_2_. Then, 100 μL 96% ethanol was used to dissolve the resulting formazan crystals and 50 μL of the formed solution was poured into the new plate wells. Later, the optical density (OD) of the solvent and specimens was measured spectrophotometrically at 570 nm. A spectral scanning multimode reader (Varioskan Flash, Thermo Scientific, Waltham, MA, USA) was applied for this purpose. The difference between the specimen and the mean of solvent OD was computed. The obtained OD is directly proportional to the number of viable cells. In order to compare assays, the OD, equal to the count of viable cells grown on each specimen, was described with the negative control group. The control group was determined to be the OD, which described the number of viable cells grown on a plate well surface. For the cytotoxicity evaluation, the ratio OD of the specimen/OD of the control group at 12 h shows if the surface of a specific material inhibits the cell growth (the ratio is below 1) or, on the contrary, promotes cell proliferation (the ratio is higher than 1).

### 2.7. Statistical Analysis

Statistical analysis was accomplished using R i386 4.0.0 (Lucent Technologies, Auckland, New Zealand). All graphs were plotted using the ggplot2 library (Lucent Technologies, Auckland, New Zealand).

Data normality was tested using the Shapiro–Wilk test, and parametric methods were used since the data showed normal distribution. Equality of variance was also tested, allowing the usage of ANOVA analysis in this study. A two-sample *t*-test was used to compare the surface roughness of the two materials.

The statistical significance level was set at *p* < 0.05. A Tukey post hoc test was used to detect the between-group difference when significant results from the ANOVA analysis were observed.

## 3. Results

### 3.1. Surface Roughness

**Figure 4:**
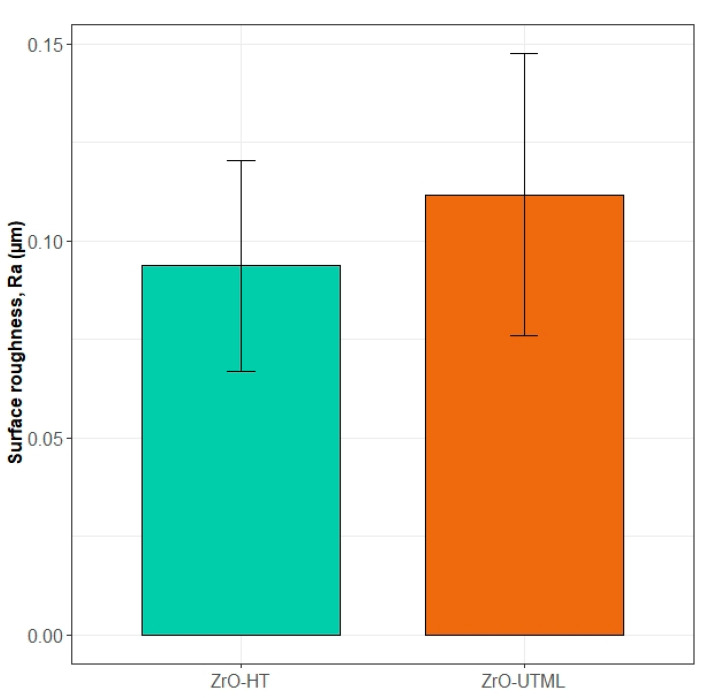
Surface roughness of the two materials included in the study.

The results from the profilometry measurements (in Ra) were 0.094 ± 0.027 μm and 0.11 ± 0.036 μm for the ZrO-HT group and ZrO-UTML group, respectively. The Shapiro–Wilk normality and the homogeneity of variance tests (*p* > 0.05) allowed for evaluating any potential differences in the studied parameter using a *t*-test. The test showed no significant difference between the materials included in the study (*t*(18) = 1.29, *p* = 0.217). A graphical representation of the results is depicted in Figure 4. Thus, surface roughness can be assumed equal in all subsequent tests.

### 3.2. Water Contact Angle

Hydrophilicity was assessed by measuring the water contact angle in this study. Mean values for the different groups tested are presented in Table 3.

The Shapiro–Wilk normality test and homogeneity of variance allowed for an ANOVA test usage (*p* > 0.05). UV-treated groups showed slightly increased means of contact angles compared to untreated materials. A one-way ANOVA test was used to assess the differences between the groups. The results did not reveal significant differences (F(16) = 3.50, *p* = 0.292).

### 3.3. Human Gingival Fibroblast (HGF) Cytotoxicity

HGF cytotoxicity was assessed as a ratio of cell viability with a control group (value = 1) after a 24 h incubation period (Figure 4). A one-way ANOVA test was used to compare the performance of the different groups. Nevertheless, values obtained for the UV-treated groups were higher within the same material.

### 3.4. Human Gingival Fibroblast (HGF) Proliferation

**Figure 5:**
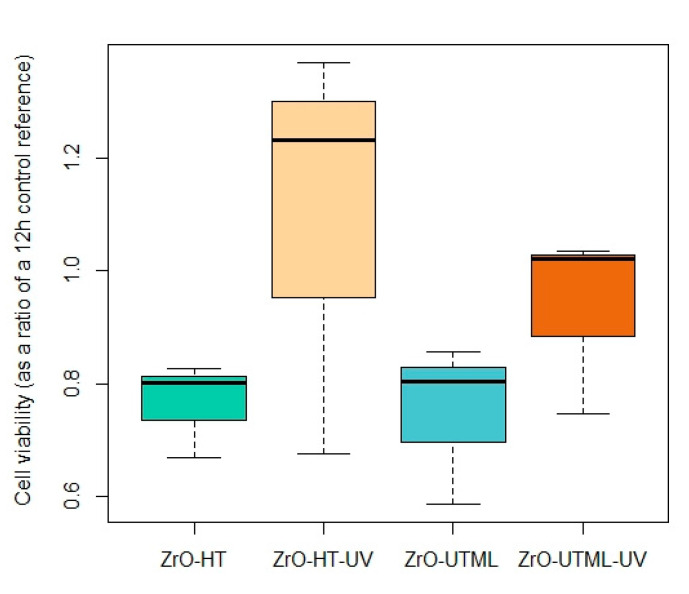
Cytotoxicity assessment as a ratio of the 12th hour control reference for the four groups included in the study.

**Figure 6:**
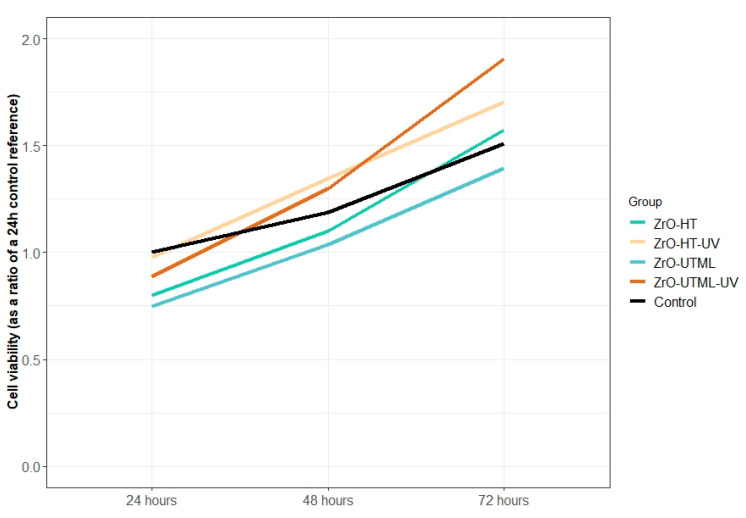
The tendency of HGF proliferation in the different study groups for the tested time-points at 24, 48, and 72 h.

HGF proliferation tended to increase over time, irrespective of the material or the UV surface treatment (Table 4 and Figure 5 and Figure 6). The highest ratio obtained for the 24 and 48 h time points was in group ZrO-HT-UV and the 72 h time point for ZrO-UTML-UV. The lowest ratio value observed for all three time points was in the ZrO-HT group.

Significant differences between groups were detected only at the 24 h time point (F(8) = 9.58, *p* = 0.005). The performed post hoc Tuckey analysis revealed a significant difference between ZrO-HT and Zr-HT-UV (*t* = 3.875, *p* = 0.020), as well as between ZrO-UTML and ZrO-HT-UV (*t* = 4.976, *p* = 0.005). At the 24 h time point, all values for the tested materials were lower than the control.

## 4. Discussion

This study evaluated the effect of UV photofunctionalization applied to two different ZrO_2_-based materials for abutment fabrication. Surface roughness, hydrophilicity, cytotoxicity, and proliferation of HGF fibroblasts were analysed. Zirconia-based materials have shown superior properties related to biocompatibility compared to all other materials currently in clinical use [22].

This study aimed to evaluate the effect of UV treatment of test specimens made of two different ZnO_2_-based ceramic materials on the hydrophilicity, cell cytotoxicity, and proliferation of human gingival fibroblasts (HGFs). The results revealed no significant difference in the hydrophilicity, cytotoxicity, and proliferation at 48 and 72 h. Thus, the null hypothesis was accepted. At 24 h, HGF proliferation showed a significant difference only in the ZrO-HT vs ZrO-HT-UV group. Hence, the alternative hypothesis was accepted. Another significant difference between the study groups, as detected by the Tuckey HDS post hoc analysis, was between the groups ZrO-UTML and ZrO-HT-UV.

Surface morphology and roughness significantly influence bacterial and cell adhesion to abutment materials [14]. In the present study, surface finishing protocols recommended by the manufacturer were applied for the included materials. Furthermore, a cleaning procedure in an ultrasonic bath in deionised water for 1 h was also performed. Finally, a non-contact profilometry was utilised to evaluate the micro-surface characteristics. Average surface roughness is classified into four categories: smooth, minimally rough, moderately rough, and rough [26]. In addition, there is variance in the measurement methodology for this parameter, including contact or non-contact profilometry, confocal microscopy, and SEM [15,23,24,25,27,28,29,30]. In the present study, minimally rough surfaces (Ra < 0.5 μm) were achieved with negligible differences in roughness, allowing for the direct comparison of both materials, disregarding these parameters’ influence on the results of subsequent analyses.

Among different surface modification methods, the photofunctionalization of Zirconia-based implant abutments has increased healing potential, improved wettability, and enhanced biological seal [24,30,31]. Three main photofunctionalization methods are used based on the radiation wavelength: UV-A (100–400 nm), UV-B (290–320 nm), and UV-C (100–290 nm) (Flanagan2016.32). In addition, mixed protocols have been used in previous studies [30]. The irradiation times vary considerably in the literature, ranging from as little as 10 min to more than 24 h [15,24,30,31,32]. In the current study, UV-C irradiation was used.

Hydrophilic surfaces are favourable for HGF adhesion, which suggests a formation of a good peri-abutment soft tissue seal [18]. Moreover, hydrophobic surfaces are associated with increased bacterial adhesion and accumulation of biofilm, slowing healing processes [33,34]. Previous studies have reported different contact angles evaluating Zirconia surfaces after photofunctionalization [20,27]. UV treatment has been shown to reduce carbon and increase oxygen and Zirconia surface content as well as lower the WCA [24]. Since the effect of photofunctionalization is time- and dose-dependent, the average change in WCA varies considerably based on the specific experimental setting. Most studies reported a significant decrease in water contact angles after photofunctionalization. Att et al. communicated the highest untreated (100.1°) and lowest UV-treated (20.8°) WCA scores. Other studies reported milder but significant increases in hydrophilicity ranging from a 10° to 50° WCA change [27,28,30]. The UV-photofunctionalization protocol used in this study did not significantly change the hydrophilicity parameters. Furthermore, an increase in the water contact angle was observed, albeit insignificant. The current study used a very thorough and detailed specimen cleaning protocol which reduced surface impurities, thus likely reducing the effect of UV towards the WCA.

During the soft tissue healing process around implant abutments, the adhesion proliferation and contact of HGFs to the materials’ surface would affect the biological seal. Furthermore, it was found that the inner surface adjacent to the biomaterial has a higher fibroblast count [35]. It was suggested that conditioning abutment surfaces with UV light would affect hydrophilicity, reduce the hydrocarbon content, and enhance surface electro-positivity, promoting protein adsorption and cellular adhesion [24,36]. The results of this study can partially confirm these suggestions. The change in the 24–48–72 h proliferation curve for HGF between the UV-treated and non-treated groups suggests that the former performs better. These results might be due to the decontamination properties of UV irradiation or the type of material used, since most other factors have been eliminated as covariates [24]. This is also evident in the cytotoxicity test where a notable difference between the UV-treated and non-treated groups was observed for both tested materials, albeit a not significant one.

This study has several limitations. All experiments were performed in vitro in sterile and well-controlled conditions using an HGF monoculture. In a clinical setting, the abutment surface is contaminated during placement and subjected to microbial adhesion and contact with inflammation fluids, blood, and different types of cells, and the study design does not include these conditions. Furthermore, the macroscopic design of the specimens and test assembly deviates from the clinical conditions. Thus, a 2D experimental model was used instead of a 3D (organotypic) model [30,37]. As changes can further influence design parameters and materials, further investigation is appropriate to assert the current results and provide clinical recommendations. The authors should discuss the results and how they can be interpreted from the perspective of previous studies and the working hypotheses. The findings and their implications should be discussed in the broadest context possible. Future research directions may also be highlighted.

## 5. Conclusions

Within the current study’s limitations, it can be concluded that in an in vitro test setting including specimens produced from ZrO_2_-based materials with insignificant differences in micro-roughness, UV surface photofunctionalization alters the HGF cell viability for the studied period up to 72 h. This change is material-dependent, and there is no significant change in the other studied parameters. Thus, UV-C irradiation of Zirconia surfaces might produce favourable results for cell proliferation.

## Figures and Tables

**Table 1:** Materials, abbreviations used in the text, and their respective manufacturer in the present study.

Abbreviations Used in Text	Material	Tradename	Manufacturer
ZrO-HT	High-translucent Zirconia	Katana^TM^ HT	Kuraray Noritake
ZrO-UTML	Ultra-translucent Multi-layered Zirconia	Katana^TM^ UTML	Kuraray Noritake

**Table 2:** Polishing protocol used in the study.

Material	Polishing Protocol Used			
ZrO-HT ZrO-UTML	MPF Zmax disc (Item No. 120-0001 Zmax Large Disc 22 × 4.5 mm) 5000–10,000 min^−1^/30 s MPF Brush Co., Nicosia, Cyprus	Edenta (R1530HP) StarGloss pink polisher for ceramics 5000 min^−1^/30 s EDENTA AG, Au/St. Gallen, Switzerland	Edenta (R1540HP) StarGloss green polisher for ceramics 5000 min^−1^/30 s EDENTA AG, Au/St. Gallen, Switzerland	Zircopol polishing paste and narrow brush 10,000 min^−1^/30 s Feguramed GmbH, Buchen, Germany

**Table 3:** Water contact angle in degrees for the four groups is presented as means ± standard deviations.

Study Group	Mean	SD	F(Df)	*p*
ZrO-HT	69.9	6.4	3.50(16)	>0.05
ZrO-HT-UV	73.7	13.9		
ZrO-UTML	79.5	12.8		
ZrO-UTML-UV	83.4	11.4		

**Table 4:** Fibroblast proliferation results are presented as mean and SD values for the three investigated periods.

Group	Viability at 24 h	Viability at 48 h	Viability at 72 h
ZrO-HT	0.80 ± 0.08	1.10 ± 0.17	1.58 ± 0.32
ZrO-HT-UV	0.98 ± 0.02	1.35 ± 0.23	1.70 ± 0.39
ZrO-UTML	0.75 ± 0.06	1.04 ± 0.17	1.39 ± 0.42
ZrO-UTML-UV	0.89 ± 0.05	1.30 ± 0.32	1.91 ± 0.36
Control	1	1.19 ± 0.02	1.51 ± 0.32

## Data Availability

Not applicable.
